# Analysis of 190 Female Patients after Appendectomy

**DOI:** 10.1155/2021/8036970

**Published:** 2021-11-27

**Authors:** Abdulrahman M. Alotaibi, Leena H. Moshref, Rana H. Moshref, Lina S. Felemban

**Affiliations:** ^1^Department of Surgery, Faculty of Medicine, University of Jeddah, Jeddah, Saudi Arabia; ^2^Department of Surgery, Doctor Soliman Fakeeh Hospital, Jeddah, Saudi Arabia; ^3^Department of Surgery, National Guard Hospital, Jeddah, Saudi Arabia

## Abstract

This study is a retrospective cohort review carried out at a single, private tertiary center. We included 190 female patients who underwent surgery for acute appendicitis between January 2016 and December 2018. Two groups of patients were analyzed based on the pregnancy. The main outcome measures were complication rate and risk of abortion during or after surgery. Out of 190 female patients, eight of them were pregnant (4.2%). The pregnant group more significantly underwent ultrasound investigation compared to the nonpregnant group. Complicated appendicitis present in two pregnant patients at advanced gestational age was not statistically significant from nonpregnant. Laparoscopic appendectomy was performed in 6/8 (75%) of pregnant compared to 158/182 (87%) in nonpregnant (*p* = 0.415). Compared to the nonpregnant, the pregnant group has a more fecolith, positive peritoneal fluid culture, and wound infection, with *E. coli* more frequently isolated in 25%. None of the pregnant patients had an abortion, preterm labor, or mortality during or after surgery. In conclusion, laparoscopic appendectomy is a low-risk operation for pregnant with acute appendicitis.

## 1. Introduction

Acute appendicitis (AA) is estimated to be one of the most frequent surgical emergencies that requires urgent intervention [1]. The stated incidence ranges 28%, 44%, and 24% in the first, second, and last trimester [2]. The clinical presentation is nonspecific, including abdominal pain, nausea, vomiting, fever, and leukocytosis, which are not helpful as predictive indices [3]. The diagnosis of AA is troublesome throughout the pregnancy due to physiological and anatomical shifts, which defer the management [4]. Early diagnosis and intervention, either surgically or by antibiotics, are vital as noncomplicated AA can rupture and lead to perinatal and maternal complications, for instance, premature delivery, miscarriage, and fetal loss [5]. This study aimed to evaluate the clinical presentation and outcome of appendectomy in pregnant and nonpregnant female patients.

## 2. Materials and Methods

This study is a retrospective review of appendectomy procedures at Dr. Soliman Fakeeh Hospital (Jeddah, Saudi Arabia). Four hundred fifty-one consecutive adult patients underwent surgery for acute appendicitis between January 2016 and December 2018. We excluded pediatric age, male patients, and appendectomy as combined with other operations. A total of 190 females were in the current study. Two groups, pregnant and nonpregnant, were diagnosed based on clinical examination, radiological imaging, and diagnostic laparoscopy. The primary outcome is the rate of complication, abortion, or mortality during or after appendectomy.

The Institutional Review Board of Fakeeh College for Medical Sciences (FCMS) approved the study protocol.

### 2.1. Surgery Techniques

Laparoscopic appendectomy is the procedure of choice in our institution to manage acute appendicitis. The decision to perform an open appendectomy or convert depends on the surgeon's preference and the patient's clinical condition.

### 2.2. Statistical Analyses

Fisher's exact test with two-sided verification or an unpaired Student's *t*-test compares the demographic and clinicopathological variables of the two groups. A *p* value of less than 0.05 indicates statistical significance. SPSS software (version 25, SPSS Inc., Chicago, IL) analyses the data.

## 3. Results and Discussion

### 3.1. Characteristics of the Pregnant and Nonpregnant Group

Among the 190 females enrolled in the study, eight of them were pregnant (4.2%). The patient's age, comorbidities, diabetes mellitus, abdominal pain duration, complicated appendicitis, appendix diameter, leukocyte count, C-reactive protein, surgical approaches, operative time, pathology finding, length of stay, and mortality were statistically insignificant among both groups (Table 1).

Compared to the nonpregnant, the pregnant group underwent more ultrasound images for evaluation (36.8 vs. 100%, respectively, *p* = 0.001) and had more positive peritoneal fluid culture (8.3 vs. 37%, respectively, *p* = 0.017) with *E. coli* and ESBL more frequently isolated.

Additionally, the wound infection rate was more significant (0.5 vs. 12.5%, respectively,*p* = 0.013), along with the fecolith as the cause of appendicitis (28.5 vs. 75%, respectively,*p* = 0.011).

### 3.2. Abortion, Mortality, and Morbidity Outcomes

In the first, second, and third trimesters, the pregnant patients were four, three, and one, respectively.

Two pregnant patients diagnosed with complicated appendicitis in the second trimester (26^th^ week) and last trimester (27^th^ week) were not significantly different from nonpregnant (25 vs. 9.5%, *p* = 0.156). There was no difference in appendicitis incidence concerning trimester; however, the complicated one occurred later in pregnancy.

None of the pregnant patients had an abortion, preterm labor, fetal loss, or mortality during or after surgery. During the follow-up period of the pregnancy, none of them developed any complications related to the operation—further detailed about pregnant group trimesters in Table 2.

## 4. Discussion

AA represents 65% of nontraumatic emergencies with a prevalence of 0.1 to 0.2% throughout all trimesters [6, 7]. In our series, four patients out of eight were in their second trimester, similarly to others who reported the second trimester as the most frequent months to have AA [8–10]. Others stated the first trimester as the most common [11, 12]. A Swedish study linked reduced numbers of AA in the 3^rd^ trimester to positive effects of hormones and TH1-mediated inflammatory response [13]. The majority of AA during pregnancy needs surgical intervention [14]. In agreement with others, the mean age of presentation is 31 years [9, 15], contrary to a younger age in China, India, and Korea [10, 16–19]. The comorbidity in some reviews affects the surgery approach, while others showed no correlation [20, 21].

The clinical and radiological evaluation of AA during pregnancy is challenging [22]. In a review of 21 pregnant, abdominal pain was higher in the first and second trimester by 57% and 23%, respectively [23]. Vomiting was the most common symptom noted in other series [9, 24]. The use of the Alvarado score in pregnancy showed positive predictive values of almost 90%, 80%, and 75% in the first, second, and third trimester [9, 22]. Our data observation has no statistical significance in both groups' WBC, neutrophil, and CRP levels, comparable to other reviews [7, 25, 26].

The ultrasound (US) is utilized in around 70% of pregnant compared to 30% in nonpregnant [15, 19, 27–29], which is imminent to our result (100 vs. 36%, *p* = 0.001, respectively). The US assisted in the diagnosis more in the first trimester than the last one (*p* = 0.004) with sensitivity and specificity of 80% and 92%, respectively [30]. According to numerous studies, CT scan was utilized in 3% of pregnant and 15% of nonpregnant women [10, 15, 28]; however in our study, nearly 30% of nonpregnant group underwent a CT scan. Magnetic resonance imaging (MRI) is a valid and safe option to diagnose despite cost disadvantages [9, 29]. An abdominal CT might be requested when the US cannot visualize the appendix in the third trimester [11].

The rate of open appendectomy (OA) vs. laparoscopic appendectomy (LA) varies between researchers. Approximately 80% of pregnant women undergo procedures in an emergency setting, with 38.4% undergoing OA vs. 63.6% LA [16]. Others reported a high rate of OA in pregnant women, which reached 73% and 92% [8, 19, 23]. From the New York State database which included 1000 patients, OA was performed in community centers by 50% and LA in academic centers by 60% [21]. In one tertiary hospital, the rate of LA was 1 out of 450 [31]. In our center, laparoscopy was the most used technique as it was performed in an academic hospital by 75% in pregnant and 85% in nonpregnant. Surgeon preference, as well as the gestational age, can affect the surgical approaches [26, 32]. Conversion to open depends mainly on intraoperative findings rather than surgeon experience [24]. The incision in OA differed in the literature with 66–93% McBurney's point, 28.6% right paramedian, and 6.6% midline laparotomy [10, 11, 23, 33].

Several management strategies are in the literature. A nationwide Japanese database exhibits conservative management of AA in 67% and surgery in 33% [29]. In a multicenter review, pregnant women were more likely to be treated with antibiotics alone (15% vs. 4%; *p* = 0.008) and to have complicated appendicitis [15]. In another review of six appendectomies in pregnant women, one case complicated by chorioamnionitis and miscarriage at 20 weeks of gestation and two patients by right iliac fossa abscesses requiring percutaneous radiological drainage, in which one of these women delivered a healthy term baby, and the other had chorioamnionitis and preterm delivery at 34 weeks, followed by neonatal death [34]. Nonetheless, early treatment improves maternal and fetal outcomes, with the average time between diagnosis and operation times less than 48 hours [11, 29]. A study from China to see the association between antibiotics and complication rate among 54 pregnant women showed that the patients with antibiotics (*n* = 34) experienced no complications. However, recurrence occurred in the conservative group in one patient during pregnancy and two patients after delivery, treated with appendectomy [35]. In our data, complicated appendicitis was 25% in pregnant vs. 9% in nonpregnant women. The majority had suppurative appendicitis and tended to have more fecolith by 75% vs. 28% (*p* = 0.011)—the conservative approach not implemented to our patients. The explanation for using antibiotics alone in some centers is that up to one-third of the patients can have a normal appendix. On the contrary, two-thirds can have appendiceal inflammation and one-fourth with abscess [10, 13, 18, 23, 26, 27, 31, 32].

Prolonged exposure to anesthesia can have an adverse effect. The mean operating time of LA is 45 minutes (range: 10–70 minutes) [17, 18, 27, 32, 35, 36]. In our data, the average time did not differ in the two groups, with a mean of 50 minutes. LA showed significantly shorter operation time, hospital stay, and earlier recovery of gastrointestinal function when compared with the OA group [8, 20]. In complicated appendicitis, the laparoscopic approach is safe in pregnancy [20]. The hospital stays in the LA group are significantly shorter, with a mean of 2.7 days (ranged 1.5–11 days) [17, 19, 21, 25, 29, 32]. Some favored LA in the first and second trimesters and OA in the third trimester, where the last trimester patients had double the fold of readmission [21].

A meta-analysis in 2018 and 2021 decided that the laparoscopic approach had a higher rate of fetal demise before 2010 [37, 38]. A study in 1999 of 700 patients showed that rates of fetal losses were 30% and 10% in the first and second trimesters, respectively [7]. A systematic review of 637 patients from 1990 to 2017 revealed 6% fetal losses in the laparoscopic procedure [39]. In our results, we had no infant or maternal mortality in both LA and OA groups. Similarly, there have been no deaths [8, 17, 18, 31, 32]. On the contrary, a meta-analysis of 17 observational studies showed that LA had a higher risk of fetal loss (5.69% vs. 3.73%) but a low preterm delivery (2.84% vs. 8.99%) [40]. Nearly one out of twenty women who underwent appendectomy can have a complicated obstetrics course in the form of preterm labor, cervical incompetence, vaginal infection, and sepsis [41]. In the last trimester of pregnancy, laparoscopic surgery is feasible and safe with acceptable risk to the fetus and mother [42, 43]. During pregnancy, the altered immune system makes the patient amenable to disseminating infection. The laparoscopic approach had decreased prevalence of wound infection when compared to open [37, 38]. Wound infection occurs in 15% of the patients who underwent appendectomy during cesarean section [10, 13, 28, 36].

The limitation of the study is a retrospective review and a low sample size of the pregnant group.

## 5. Conclusions

Appendectomy during pregnancy carries a low risk regarding fetal abortion or mortality—no significant difference between the pregnant and nonpregnant group concerning appendix perforation, delayed diagnosis, or complications. More evidence is obliged to investigate the advantages of conservative management, such as antibiotics, for acute appendicitis during pregnancy.

## Figures and Tables

**Table 1:** Demographics and clinicopathological features of the study patients according to pregnancy status.

Variables	Values as mean ± SD or no. of patients (%)		*p* value^a^
	Nonpregnant group (*n* = 182)	Pregnant group (*n* = 8)	
Age at surgery, years	29 ± 11.3	31 ± 4.8	0.65
*ASA score*			0.005
I	81 (44.5)	1 (12.5)	
II	99 (54.4)	6 (75)	
III	1 (0.55)	1 (12.5)	
IV	1 (0.55)	0 (0)	

Length of admission	2.1 ± 1.3	2.3 ± 1.0	0.68
Diabetes	10	0	0.64
Comorbidities (DM not included)	32	2	0.43

*Pain duration*			0.54
Less than 24 h	81	2	
24–48 h	69	4	
More than 48 h	31	2	

WBC (u/L)	11.71 ± 4.2	14.51 ± 4.2	0.068
Neutrophil (%)	71.65 ± 14.3	82.36 ± 9.2	0.052
CRP (mg/L)	42.51 ± 60.9	68.90 ± 64.4	0.26

*Type of radiology study*			0.001
Ultrasonography	67	8	
CT scan	112	0	
Appendix diameter (mm)	10.48 ± 3.3	10.33 ± 1.36	0.91

*Complicated appendicitis*			0.18
Yes	17 (9.30)	2 (25)	
No	162 (90.7)	6 (75)	

*Operative approach*			0.08
Open	23 (12.6)	2 (25)	
Laparoscopy	158 (87.4)	6 (75)	
Operative time (min)	48 ± 19.6	55 ± 22.7	0.33

*Peritoneal fluid culture*			0.017
Positive	15 (8.3)	3 (37.5)	
Negative/not done	167 (91.7)	5 (62.5)	

*Postoperative complications*			0.013
No	175 (96.2)	7 (87.5)	
Wound infection	1 (0.5)	1 (12.5)	
Collection	4 (2.2)	0	
Nonsurgical	2 (1.1)	0	

*Fecolith*			0.011
Yes	52 (28.5)	6 (75)	
No	130 (71.5)	2 (25)	

*Pathology*			0.795
No suppuration	39 (21.4)	1 (12.5)	
Acute suppuration	123 (67.6)	5 (62.5)	
Gangrenous	4 (2.2)	1 (12.5)	
Perforated	1 (0.55)	1 (12.5)	
Endometriosis	1 (0.55)	0	
Carcinoid	2 (1.1)	0	
Chronic appendicitis	10 (5.5)	0	
Granulomatous	2 (1.1)	0	
Abortion	0	0	
30-day mortality	0	0	

^a^Pearson's chi-square test. SD: standard deviation; ASA score: American Society of Anesthesiologists score; CRP: C-reactive protein.

**Table 2:** Rate of complicated appendicitis based on trimester.

Pregnancy in weeks	Noncomplicated (*n* = 6)	Complicated (*n* = 2)	*p* value^a^
			0.33
6	1	0	
8	1	0	
10	1	0	
13	1	0	
17	1	0	
24	1	0	
26	0	1	
27	0	1	

^a^Pearson's chi-square test.

## Data Availability

The data used to support the findings of this study are available upon request from the corresponding author Dr. Abdulrahman Alotaibi, Department of Surgery, Faculty of Medicine, University of Jeddah, Saudi Arabia (e-mail: aalotaibi@uj.edu.sa).
